# Optimal body mass normalization of power output for accurate prediction of estimated cycling performance over complex time-trial courses

**DOI:** 10.3389/fspor.2025.1599319

**Published:** 2025-08-18

**Authors:** Marton Horvath, Erik P. Andersson

**Affiliations:** Department of Health Sciences, Swedish Winter Sports Research Centre, Mid Sweden University, Östersund, Sweden

**Keywords:** allometric scaling, critical power, numerical methods, performance prediction, power-duration relationship, sports engineering, time-trial performance

## Abstract

**Introduction:**

Power profiling is widely used in cycling performance analysis, but both absolute and mass-normalized power outputs have limitations as performance indicators, as they neglect external factors such as terrain, wind, aerodynamic drag, and pacing strategy. To address these limitations, this study introduced a numerical method to quantify how external forces acting on the cyclist influence the conversion of power output into race velocity. Thus, the study aimed to enable accurate prediction of cycling performance based on estimated mean power output over complex time-trial courses.

**Methods:**

Time-trial performances of five elite-level road cyclist profiles—a sprinter, climber, all-rounder, general classification (GC) contender, and a time trialist—were estimated using the power-duration relationship and previously published normative data. These performance estimates were applied to both simplified hypothetical courses and complex real-world time-trial courses. Optimal mass exponents for the power-to-mass ratio were determined based on the estimated average speeds over the respective course sections, cyclist morphology, and external factors such as gradient and wind velocity.

**Results:**

Across two recent Grand Tour individual time-trial courses, stage 21 of the 2024 Tour de France and stage 7 of the 2024 Giro d’Italia, the duration-weighted optimally mass-normalized power output metrics were $\text{W}/{\text{kg}}^{0.6068}$ and $\text{W}/{\text{kg}}^{0.4891}$, respectively. These metrics accurately predicted the estimated performances of the five defined cyclist profiles ($R^{2}=0.99$ for both).

**Discussion:**

The results indicate that the duration-weighted optimal mass exponents for the power-to-mass ratio are course-specific. By deriving optimal mass exponents across various modeled courses and wind conditions, the study was able to precisely quantify the influence of road gradient, headwind speed, and bicycle mass on the conversion of power output relative to body mass into speed. Further research is needed to validate the presented method for determining optimal mass exponents in real-world performance settings.

## 1 Introduction

Strong time-trial performances are crucial for success in the general classification (GC) of cycling Grand Tours (1). Thus, identifying the optimal balance between body mass and power output is particularly challenging, as GC-contenders must perform well in both climbing stages, where a high body-mass-normalized power output is essential, and flat time trials, which require high absolute power output. Therefore, accurately predicting time-trial performance is highly relevant in professional cycling, with direct implications for training optimization and tactical decision-making.

Power profiling in cycling involves the assessment of power outputs using power meters over various durations during training and competition (2). This practice is fundamentally tied to the power-duration relationship, which allows for predicting performance across different exercise durations (3). The two most commonly used power metrics for establishing cyclists’ power output profiles and assessing their performance potential are absolute power output (i.e., W) and power output normalized to body mass in kilograms (i.e., W/kg) (4–10). However, these metrics alone do not accurately predict a cyclist’s true performance capacity (11). For example, between two cyclists with the same body-mass-normalized power output (i.e., power-to-mass ratio) over a given exercise duration, the heavier cyclist will reach a higher speed on flat terrain. Conversely, if two cyclists have identical absolute power output, the lighter cyclist will have an advantage on the uphill sections of the competition course [see Swain (12) for further details]. To address this limitation, prior studies have developed allometrically scaled power metrics based on empirical performance tests (11–15). However, none of these former studies have presented a precise method for predicting performance on courses with variable terrain or wind exposure.

The present study aimed to develop a method for quantifying how external forces determine the power output-body mass ratio, which accurately reflects cycling performance. Additionally, the study was designed to examine how these ratios were influenced by internal factors such as drag area, power output, and equipment mass, as well as external parameters such as gradient and wind.

## 2 Materials and methods

### 2.1 General overview

**Figure 1:**
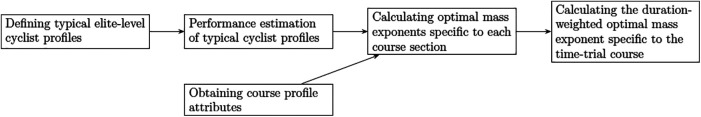
Schematic overview of the developed process for deriving optimally normalized power output metrics, enabling accurate performance prediction on complex individual time trial (ITT) courses.

This study presents a novel numerical approach for deriving the optimal body mass exponent in the power-to-mass ratio for performance prediction, determined by the primary resistive forces acting on a cyclist. The approach utilized the power-duration relationship to estimate time-trial performance across five typical elite-level road cyclist profiles, constructed using previously published normative data, over both simplified hypothetical and complex real-world time-trial courses. A schematic overview of the method is presented in Figure 1, and the following subsections describe each step in detail.

### 2.2 Defining typical elite-level cyclist profiles

As the first step in deriving the power-to-mass ratio resulting in optimized performance prediction, five elite-level cyclist profiles were created according to cyclist typology. These were constructed using previously published data from 144 male World Tour and Pro Continental cyclists, collected during training and competition, over several years (10). These cyclist profiles were designed to represent “typical” riders in the professional peloton based on both morphology and power profile characteristics. The cyclist profiles represented the following rider categories: sprinter, climber, all-rounder, general-classification (GC)-contender, and time trialist. These profiles were intended to reflect the key performance characteristics commonly observed among elite-level road cyclists (Table 1).

**Table 1:** Morphological characteristics and critical power model parameters—critical power (CP) and curvature constant ($W^{\prime }$)—for the defined typical cyclist profiles. Morphological data were obtained from Valenzuela et al. (10), while CP and $W^{\prime }$ values were derived from the reported power data using Equation 1. Mean values of these parameters were used in time-trial performance estimations for each model.

Type	Body mass (kg)	Height (m)	*C*_*d*_*A* (*m*^2^)	*CP* (W)	*W*' (kJ)
Climber (n=50)	63.2 (4.4)	1.77 (0.06)	0.264 (0.006)	354.1 (24.6)	23.0 (1.3)
Sprinter (n=11)	80.2 (6.5)	1.87 (0.06)	0.285 (0.007)	381.3 (23.7)	38.6 (4.5)
Time trialist (n=11)	72.6 (5.4)	1.84 (0.08)	0.276 (0.006)	395.3 (31.8)	22.0 (2.7)
GC-contender (n=7)	63.8 (4.5)	1.76 (0.07)	0.265 (0.006)	388.6 (24.0)	18.1 (2.3)
All-rounder (n=65)	69.5 (5.5)	1.81 (0.06)	0.272 (0.007)	361.1 (31.7)	30.1 (0.2)

n, sample size used for defining the respective typical cyclist profile; $C_{d}A$, drag area; CP, critical power; $W^{\prime }$, work capacity above critical power; data are represented as mean (SD).

### 2.3 Time-trial performance estimation of typical cyclist profiles

The subsequent step in the process of deriving the power-to-mass ratio reflecting cycling performance was to estimate the performance of the five defined cyclist profiles over the analysed race courses. This step was essential for estimating the power required to counteract aerodynamic drag and for calculating the time the cyclist would spend on the total course as well as on each course section. Firstly, simplified performance estimations were carried out over hypothetical flat and uphill courses representing constant inclines over a 10 km distance. For the flat conditions ($\alpha =0^{\circ }$), both windstill and headwind scenarios were modeled. As uphill conditions, moderate ($\alpha =2^{\circ }$) and steep ($\alpha =7^{\circ }$) inclines were examined. Subsequently, complex time-trial courses of two recent Grand Tours were analysed. In addition, comprehensive analyses were performed, aiming to reveal the underlying relationships between the optimal mass exponent of the power-to-mass ratio and such factors as incline, wind velocity, power output and equipment mass.

To obtain the power-duration relationship parameters of the five created typical cyclist profiles, the following critical power model was fitted to the respective power data for each cyclist profile:

(1) $$P(t)=CP+\frac{P_{\mathrm{an}}}{1+t/\tau },$$

where P is power output, CP is critical power, $P_{\mathrm{an}}$ is power capacity above CP, t is duration, τ represents the time constant corresponding to the depletion of half of $P_{\mathrm{an}}$ (i.e., $CP+P_{\mathrm{an}}/2$), and the product of $P_{\mathrm{an}}$ and τ equals the work capacity above CP (i.e., $W^{\prime }$).

Neglecting marginal losses such as frictional loss in the drive train and wheel bearings, cycling power output when traveling in a straight line at a constant speed can be expressed as:

(2) $$P=P_{\mathrm{grav}}+P_{\mathrm{roll}}+P_{\mathrm{air}},$$

where $P_{\mathrm{grav}}$, $P_{\mathrm{roll}}$ and $P_{\mathrm{air}}$ denote power against gravity, rolling resistance and aerodynamic drag, respectively and P is the propulsive power output generated by the cyclist (16). Assuming constant system mass, road conditions, no inertia and a constant drag area, expressing each component in Equation 2 results in:

(3) $$P=\left(m_{\mathrm{sys}}\;g(\sin(\alpha )+C_{\mathrm{rr}}\cos(\alpha ))+0.5\;C_{d}A\rho (v+v_{\mathrm{wind}}\;\cos(\phi ){)}^{2}\right)\;v,$$

where $m_{\mathrm{sys}}$ is the system mass, amounting to the sum of body mass (m) and equipment mass ($m_{\mathrm{equip}}$), g is the gravitational acceleration (i.e., 9.81 $m/s^{2}$), α denotes the incline of the road, $C_{\mathrm{rr}}$ is the rolling resistance coefficient, $C_{d}$ is the drag coefficient, A is the projected frontal area of the cyclist-bicycle system, ρ is the air density, the $v+v_{\mathrm{wind}}\cdot \cos(\phi )$ expression is the headwind velocity relative to the cyclist (i.e., relative velocity), where $v_{\mathrm{wind}}$ is the velocity of wind, ϕ denotes the angle of wind with the direction of travel and v is the cyclist’s speed of travel (for a more refined formulation refer to Equation A1 in the Appendix). To determine the drag area, the drag coefficient and the projected frontal area of the cyclist-bicycle system were allometrically scaled ($C_{d}A=0.0725\cdot m^{0.312}$), assuming a standard bicycle geometry, as described by Heil (17).

The mathematical model for estimating the speed (v) of the typical cyclist profiles over the hypothetical time-trial courses was derived by substituting Equation 1, into the left-hand side of Equation 3, resulting in:

(4) $$CP+\frac{P_{\mathrm{an}}}{1+\frac{s}{v\tau }}=\left(m_{\mathrm{sys}}\;g\left(\sin(\alpha )+C_{\mathrm{rr}}\;\cos(\alpha )\right)+0.5\;C_{d}A\rho {\left(v+v_{\mathrm{wind}}\;\cos(\phi )\right)}^{2}\right)\;v,$$

where s denotes course distance, while CP and $P_{\mathrm{an}}$ values corresponding to each typical cyclist profile are presented in Table 1. Thus, time-trial performance was defined as the speed corresponding to the estimated average power output over the given time-trial course, assuming an even pacing strategy. This equation was solved numerically for speed using the secant method implemented in Python v3.12.5 (Python Software Foundation, Wilmington, DE, USA). Iterations continued until the difference between successive approximations of v met the predetermined convergence criterion $|v_{k+1}-v_{k}|<10^{-8}$ m/s. Equipment mass ($m_{\mathrm{equip}}$) was set to 6.8 kg, while $C_{\mathrm{rr}}$ and ρ were set to 0.005 and 1.225 kg/m^3^, respectively, for time-trial performance estimations on Grand Tour stage courses.

### 2.4 Obtaining time-trial course attributes

To demonstrate the proposed method for deriving optimally normalized power output for complex courses, the course profiles of recent Grand Tour individual time trial stages (ITTs) were analyzed, namely stage 7 of the 2024 Giro d’Italia (Foligno—Perugia, 10th May) and stage 21 of the 2024 Tour de France (Monaco—Nice, 21st July). Both stages featured significant climbs, they measured 40.9 and 37.8 km in length, with total elevation gains of 341 and 663 m, and total elevation losses of 107 and 660 m, respectively.

**Figure 2:**
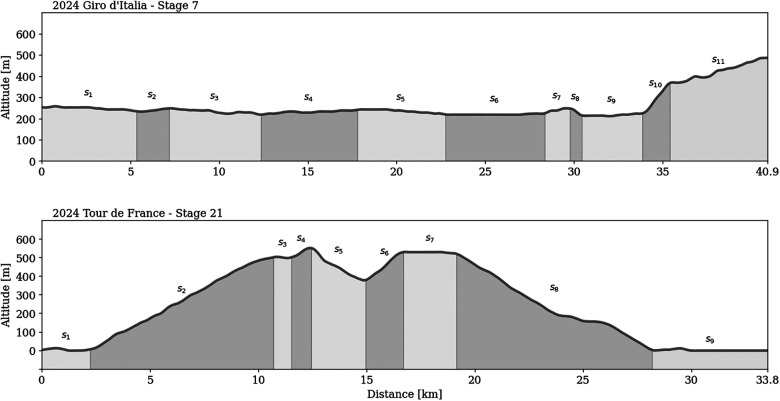
Schematic representation of the analysed Grand Tour individual time trial (ITT) courses, with course sections identified based on terrain profile. $s_{i}$ marks the ith section of the course.

To simplify time-trial performance estimations on these real-world courses, we assumed two-dimensional time-trial courses in a vertical plane, consistent with the methods used in previous studies (18–21). Course profiles were parsed using a custom-made algorithm implemented in Python v3.12.5. This process involved the identification of surface points, i.e., course coordinates, extracted from two-dimensional course profile schematics obtained online (22). The extracted coordinates were interpolated using cubic spline interpolation and filtered using a Savitzky-Golay filter, to generate the virtual course profile models used in the further analyses (23, 24). Each course profile was then divided into distinct sections concerning terrain profile (i.e., flat, uphill, and downhill sections). This process resulted in 11 and 9 sections for the Giro d’Italia and the Tour de France stages, respectively (Figure 2). Section boundaries were defined at points where substantial changes in the course inclination occurred, and attributes for each section, such as distance and elevation gain, were extracted. The duration (t) required to complete each section of the course was determined using the estimated mean speed of each typical cyclist profile over the specific section, based on Equation 4, and assuming an estimated constant mean power output across the entire course. The total finishing time was calculated as the sum of section durations.

### 2.5 Defining the optimal mass exponent of the power-to-mass ratio

When aiming to mass normalize power output for optimized performance prediction (i.e., to define the optimal mass exponent in the power-to-mass ratio), a key challenge is to establish a power metric that reflects performance independently of body mass. The underlying assumption is that, under consistent external conditions and for cycling at a given speed, the value of an optimally normalized power metric should indicate equivalent performance across cyclists of different body masses. In other words, a higher optimally mass-normalized power output should indicate superior road-cycling performance.

**Figure 3:**
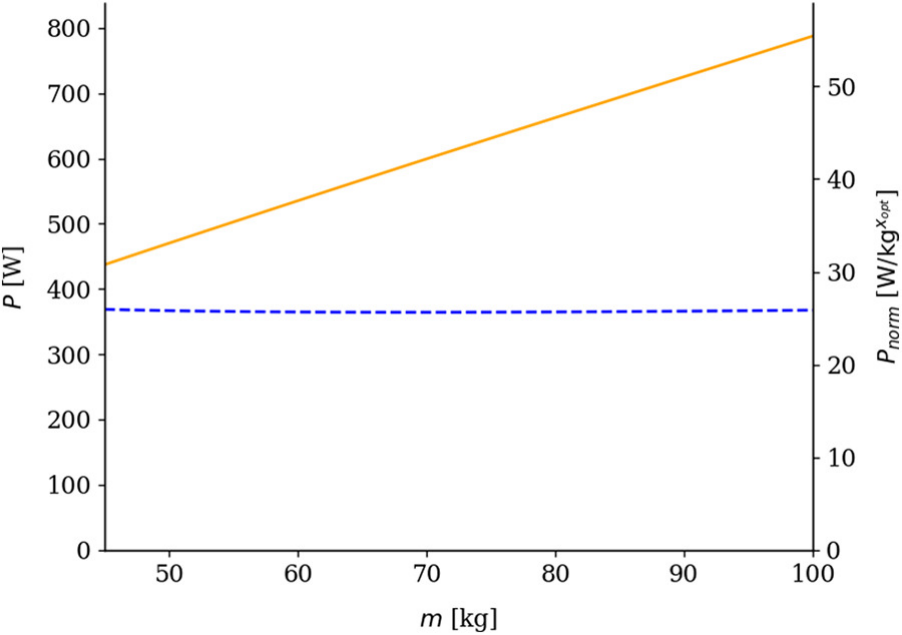
Illustration of the derived absolute power output (P) as a function of body mass (m) (orange solid curve), and its transformation into the derived power-to-mass ratio ($P/m^{x_{\mathrm{opt}}}$) (blue dashed curve). Values are shown across a body mass range of 45–100 kg, assuming constant speed (v=10 m/s) and incline ($\alpha =3^{\circ }$). The derived metric ($P_{\mathrm{norm}}=4.7\times 10^{-7}m+25.72$) demonstrates approximate body mass independence across the studied range.

To achieve this, the mass-exponent x in the power-to-mass ratio $P/m^{x}$, was determined iteratively, aiming to eliminate the effect of body size (m), such as that $P/m^{x}$ remains constant across riders of different body masses. This was accomplished by minimizing the absolute slope |a| of the regression line:

(5) $$P/m^{x}=am+b,$$

where b is the y-intercept of the regression line. The mass exponent in Equation 5 fulfilling this criterion was designated as the optimal mass exponent ($x_{\mathrm{opt}}$):

(6) $$x_{\mathrm{opt}}={\text{arg min}}_{x\in [0,1]}|a|,$$

and the slope of the regression line in Equation 6 was expressed as:

(7) $$a=\frac{\sum_{i=1}^{n}(m_{i}-\bar{m})(P_{\mathrm{norm},i}-{\bar{P}}_{\mathrm{norm}})}{\sum_{i=1}^{n}(m_{i}-\bar{m}{)}^{2}},$$

where $\bar{m}$ and ${\bar{P}}_{\mathrm{norm}}$ denotes the mean body mass and mean optimally normalized power output, respectively, and $m_{i}\in Z\cap [45,100]$ (i.e., body mass takes up all integers between 45 and 100 kg). The optimization algorithm iterated Equation 7 over each $x_{\;j}\in [0,1]$ using increments of $10^{-5}$. The algorithm tracked the slope of the regression line and its corresponding body mass exponent across all iterations. If the current $\left|a_{\;j}\right|$ that corresponded to $x_{\;j}$ was smaller than the current minimal slope corresponding to the stored exponent, this stored exponent was overwritten to $x_{\;j}$. After completing all iterations for x, the algorithm returned the value of $x_{\;j}=x_{\mathrm{opt}}$, which corresponded to the minimal absolute slope of the regression line (Figure 3).

### 2.6 Defining the duration-weighted optimal mass exponent for complex courses

For complex courses that include varying terrain types or fluctuating wind conditions, we introduced the concept of the duration-weighted optimal mass exponent (${\bar{x}}_{\mathrm{opt}}$). In practice, any time-trial course can be segmented into a discrete number of arbitrary sections (n), and the estimated performance of the typical cyclist profiles over these sections can be characterized by mean section durations $\{{\bar{t}}_{i}{\}}_{i=1,2,\ldots ,n}$. By determining the optimal mass exponent $\{x_{{\mathrm{opt}}_{i}}{\}}_{i=1,2,\ldots ,n}$ for each section, the overall course-specific exponent was calculated as the duration-weighted arithmetic mean of these section-specific exponents as:

(8) $${\bar{x}}_{\mathrm{opt}}=\frac{\sum_{i=1}^{n}x_{{\mathrm{opt}}_{i}}\cdot {\bar{t}}_{i}}{\sum_{i=1}^{n}{\bar{t}}_{i}},$$

where the denominator represents the estimated mean total finishing time of the group of typical cyclists.

## 3 Results

For the 10 km hypothetical course consisting of completely flat terrain, time-trial performance estimation yielded estimated average speeds of 12.92±0.12 m/s under windless condition (i.e., $v_{\mathrm{wind}}=0\;m/s$) and 9.87±0.13 m/s when a headwind of $v_{\mathrm{wind}}=+\;5\;m/s$ was included in the model. The corresponding optimal body mass exponents derived using these mean estimated average speeds were 0.3834 and 0.3674, respectively, indicating a decrease in the optimal mass exponent when headwind was added (Table 2). In the modeled uphill scenarios, estimated mean speeds over the 10 km hypothetical courses were 9.11±0.27 m/s for a moderate incline ($\alpha =2^{\circ }$) and 3.96±0.26 m/s for a steep incline ($\alpha =7^{\circ }$) (Table 2). The corresponding optimal body mass exponents were 0.7197 and 0.8929, respectively, demonstrating an increase with increasing gradient.

**Table 2:** Estimated average speed, finishing time and average power output of typical cyclist profiles over 10 km hypothetical courses with constant incline under varying wind conditions. Optimal mass exponents of the power-to-mass ratio ($x_{\mathrm{opt}}$), based on mean estimated average speed across typical cyclist profiles, were determined as follows: 0.3834 for a flat course ($\alpha =0^{\circ }$) with no wind, 0.3674 for a flat course ($\alpha =0^{\circ }$) with a +5 m/s headwind ($\phi =0^{\circ }$), 0.7197 for a moderate incline ($\alpha =2^{\circ }$) with no wind, and 0.8929 for a steep incline ($\alpha =7^{\circ }$) with no wind.

Conditions	Type	v (m/s)	$t_{\mathrm{est}}$ (s)	P (W)	P/m (W/kg)	$P/C_{d}A$ ($W/m^{2}$)	$P/m^{x_{\mathrm{opt}}}$ ($W/{\mathrm{kg}}^{x_{\mathrm{opt}}}$)
$\alpha =0^{\circ }$ $v_{\mathrm{wind}}=0$ m/s	GC-contender	13.11	762.5	411.9	6.46	1553	83.72
	Sprinter	12.91	775.2	429.5	5.36	1508	79.97
	Climber	12.79	782.0	382.6	6.05	1448	78.05
	All-rounder	12.81	780.8	398.3	5.73	1462	78.35
	Time trialist	13.01	768.5	423.2	5.83	1533	81.86
	Mean	12.92	773.8	409.1	5.89	1500	80.39
	95% CI	0.17	10.2	17.4	0.51	56	2.98
$\alpha =0^{\circ }$ $v_{\mathrm{wind}}=+5$ m/s	GC-contender	10.07	992.9	406.6	6.37	1533	88.32
	Sprinter	9.82	1018.2	418.3	5.22	1468	83.54
	Climber	9.74	1027.0	376.0	5.95	1422	81.96
	All-rounder	9.74	1026.5	389.7	5.61	1430	82.02
	Time trialist	9.97	1002.9	416.8	5.74	1509	86.34
	Mean	9.87	1013.5	401.5	5.78	1472	84.44
	95% CI	0.19	18.8	18.3	0.53	60	3.49
$\alpha =2^{\circ }$ $v_{\mathrm{wind}}=0$ m/s	GC-contender	9.55	1046.7	405.7	6.36	1529	20.38
	Sprinter	8.47	1143.7	414.3	5.17	1454	17.66
	Climber	9.15	1093.0	374.7	5.93	1417	18.95
	All-rounder	8.96	1116.4	387.4	5.57	1422	18.30
	Time trialist	9.17	1090.9	415.1	5.72	1503	19.00
	Mean	9.11	1098.1	399.4	5.75	1465	18.86
	95% CI	0.37	44.4	22.1	0.55	62	1.25
$\alpha =7^{\circ }$ $v_{\mathrm{wind}}=0$ m/s	GC-contender	4.36	2293.5	396.5	6.21	1495	9.70
	Sprinter	3.58	2796.9	395.0	4.93	1387	7.88
	Climber	4.05	2470.1	363.3	5.75	1374	8.96
	All-rounder	3.82	2614.3	372.5	5.36	1367	8.44
	Time trialist	3.98	2511.9	404.0	5.56	1463	8.80
	Mean	3.96	2537.4	386.2	5.56	1417	8.76
	95% CI	0.36	230.4	21.6	0.60	72	0.83

α, incline; $v_{\mathrm{wind}}$, velocity of the wind; v, estimated velocity of the cyclist profile; $t_{\mathrm{est}}$, estimated finishing time; $x_{\mathrm{opt}}$, optimal mass exponent of the power-to-mass ratio.

**Figure 4:**
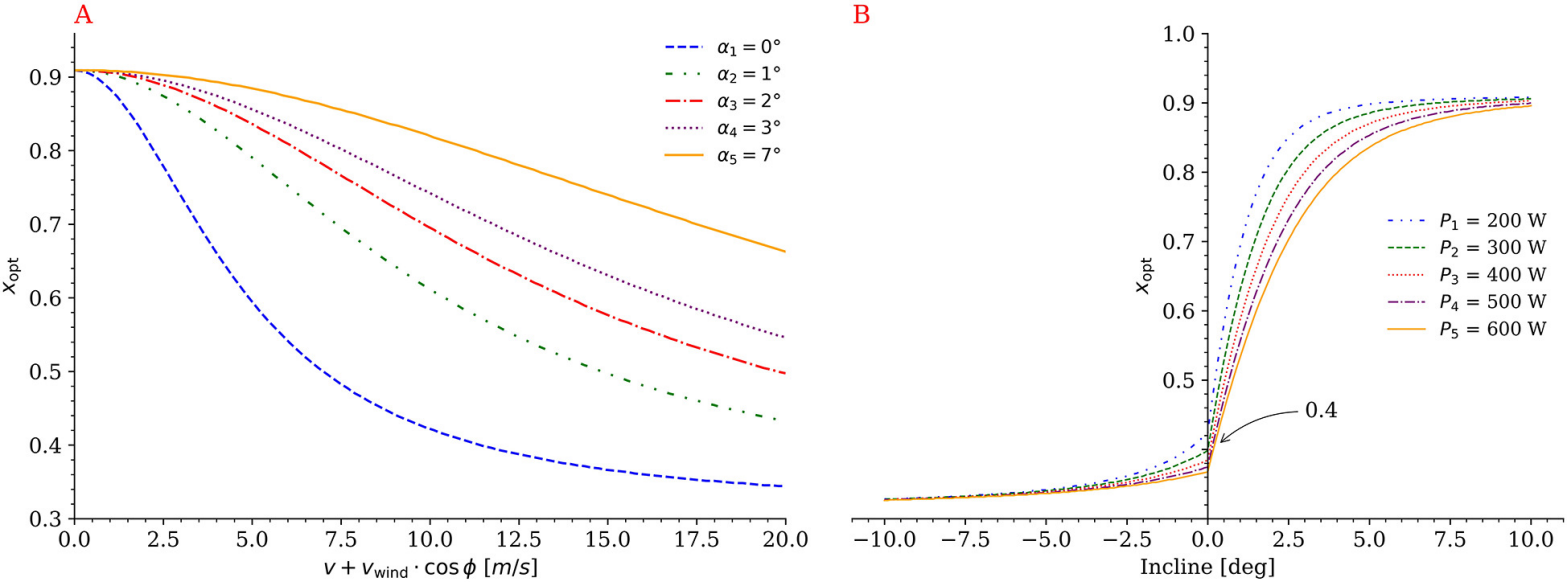
Relationships between the optimal mass exponent of the power-to-mass ratio ($x_{\mathrm{opt}}$) and relative velocity at different inclines **(A)**, and between $x_{\mathrm{opt}}$, power output, and incline **(B)**, for a cyclist with body mass m=70 kg.

Further analyses revealed that the curvature of the optimal mass exponent curve decreases as the incline increases, demonstrating that $x_{\mathrm{opt}}$ increases with steeper uphill gradients. In contrast, a higher headwind velocity relative to the cyclist ($v+v_{\mathrm{wind}}\cdot \cos\phi$) results in a lower $x_{\mathrm{opt}}$ (Figure 4A). A general pattern was also observed whereby higher-performing cyclists, that reach greater velocities due to a higher power output, exhibit lower $x_{\mathrm{opt}}$ values at a given incline. This suggests that the performance-predictive ability of mass-normalized power output (i.e., W/kg) is inversely related to performance level. As shown in Figure 4B, if curves for different constant power outputs are assumed to represent different performance levels, then drag area normalized power output becomes relatively more important at higher performance levels due to increased effective headwind. However, this effect remains relatively small.

**Figure 5:**
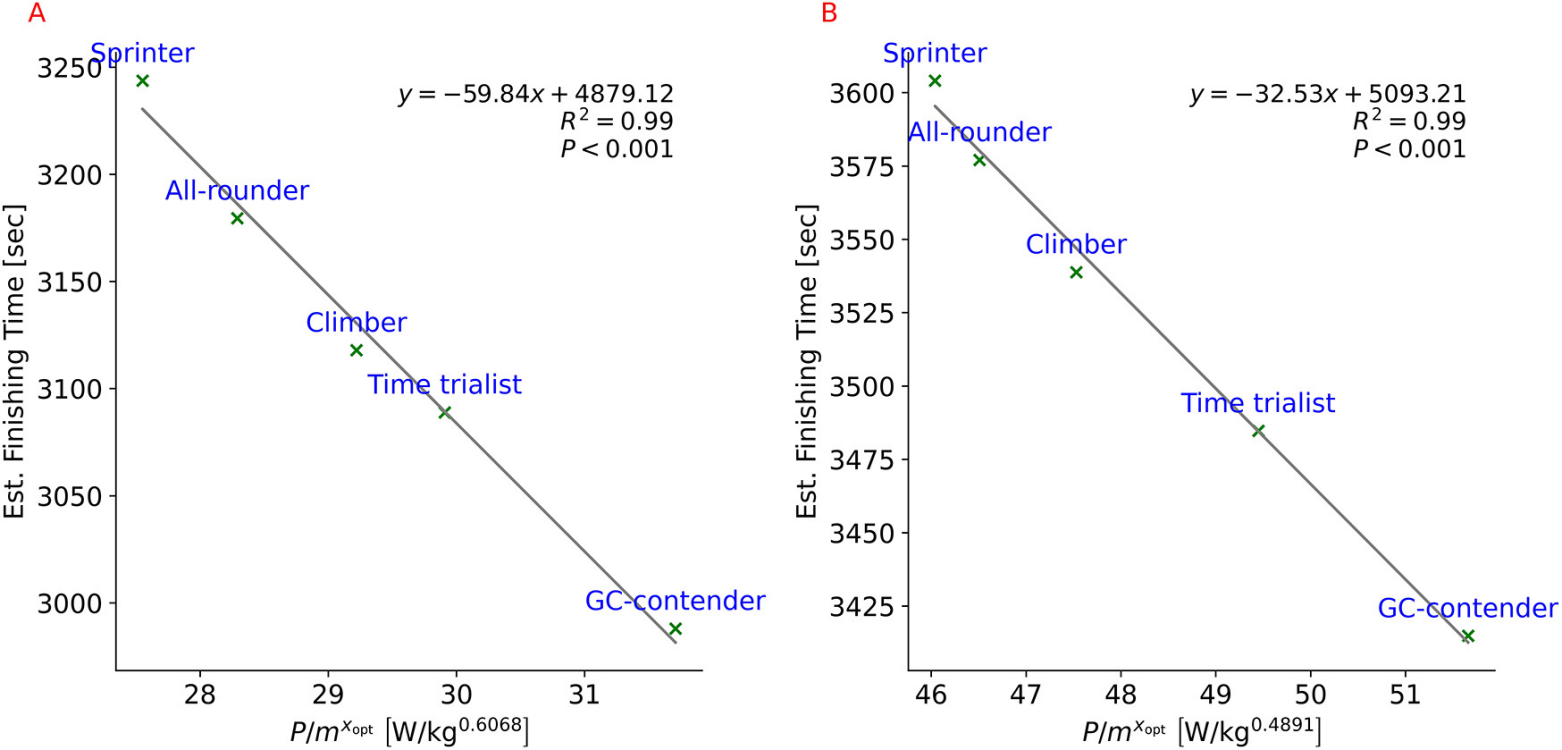
Derived power-to-mass ratios ($P/m^{{\bar{x}}_{\mathrm{opt}}}$) and total finishing times for the defined typical cyclist profiles over stage 21 of the 2024 Tour de France **(A)** and stage 7 of the 2024 Giro d’Italia **(B)**.

Based on Equation 8, the calculated ${\bar{x}}_{\mathrm{opt}}$ values for the analysed Giro d’Italia and Tour de France ITT courses were 0.4891 (95% CI: 0.4767–0.5015) and 0.6068 (95% CI: 0.5799–0.6337), respectively. These values correspond to optimal exponents expected for cycling on constant inclines of 0.44${}^{\circ }$ and 1.16${}^{\circ }$, respectively, based on the mean estimated average speeds of typical cyclist profiles over the respective courses under no-wind conditions (Tables 3, 4). Using the defined duration-weighted optimal mass exponents, linear regression analyses effectively captured variations in estimated total finishing times, demonstrating a robust ability to predict performance outcomes over the investigated time-trial courses (Figure 5).

**Table 3:** Terrain profile, estimated average speeds, and finishing times of typical cyclist profiles across the identified sections of stage 21 of the 2024 Tour de France. Optimal mass exponents of the power-to-mass ratio ($x_{\mathrm{opt}}$) were calculated for each section, and the course-specific, duration-weighted mass exponent (${\bar{x}}_{\mathrm{opt}}$) was 0.6068.

Parameters	Sections and total course									
	1	2	3	4	5	6	7	8	9	Total course
Distance (m)	2231	8369	884	1180	2494	1476	2657	8893	5579	33763
Elevation gain (m)	0.0	470.7	−7.5	52.7	−158.2	139.3	−11.3	−482.0	0.0	3.8
Incline (deg)	0.00	3.22	−0.49	2.56	−3.63	5.39	−0.24	−3.10	0.00	–
Estimated average speed (m/s)										
GC-contender	12.92	7.62	13.82	8.54	19.35	5.39	13.36	18.46	12.92	11.30
Sprinter	12.50	6.59	13.56	7.54	19.96	4.48	13.02	18.95	12.50	10.41
Climber	12.53	7.18	13.45	8.09	19.09	5.02	12.98	18.19	12.53	10.83
All-rounder	12.48	6.90	13.46	7.83	19.40	4.77	12.96	18.46	12.48	10.62
Time trialist	12.78	7.15	13.76	8.09	19.73	4.96	13.26	18.78	12.78	10.93
Mean	12.64	7.09	13.61	8.02	19.50	4.92	13.12	18.57	12.64	10.82
95% CI	0.22	0.42	0.19	0.41	0.38	0.37	0.20	0.33	0.22	0.35
Estimated finishing time (s)										
GC-contender	172.72	1097.94	63.96	138.19	128.92	273.74	198.89	481.66	431.92	2988.0
Sprinter	178.49	1269.57	65.18	156.58	124.95	329.31	204.08	469.19	446.34	3243.7
Climber	178.04	1165.06	65.70	145.83	130.68	293.82	204.65	488.91	445.23	3117.9
All-rounder	178.77	1212.15	65.68	150.75	128.58	309.64	205.03	481.83	447.06	3179.5
Time trialist	174.59	1169.87	64.24	145.80	126.41	297.61	200.38	473.46	436.58	3088.9
Mean	176.52	1182.92	64.95	147.43	127.90	300.83	202.61	479.01	441.43	3123.6
95% CI	3.01	70.39	0.90	7.56	2.49	22.77	3.09	8.61	7.53	107.1
$x_{\mathrm{opt}}$	**0.3854**	**0.8168**	**0.3764**	**0.7748**	**0.3453**	**0.8779**	**0.3814**	**0.3483**	**0.3854**	**0.6068**
95% CI	0.0191	0.0272	0.0145	0.0308	0.0199	0.0150	0.0161	0.0182	0.0191	0.0269

$x_{\mathrm{opt}}$, optimal mass exponent of the power-to-mass ratio.

**Table 4:** Terrain profile, estimated average speeds, and finishing times of typical cyclist profiles across the identified sections of stage 7 of the 2024 Giro d’Italia. Optimal mass exponents of the power-to-mass ratio ($x_{\mathrm{opt}}$) were calculated for each section, and the course-specific, duration-weighted mass exponent (${\bar{x}}_{\mathrm{opt}}$) was 0.4891.

Parameters	Sections and total course											
	1	2	3	4	5	6	7	8	9	10	11	Total course
Distance (m)	5343	1835	5182	5440	4957	5601	1416	676	3412	1545	5472	40880
Elevation gain (m)	−19.8	14.8	−28.7	23.7	−23.7	4.0	24.7	−34.6	10.9	145.3	117.6	234.2
Incline (deg)	−0.21	0.46	−0.32	0.25	−0.27	0.04	1.00	−2.93	0.18	5.37	1.23	–
Estimated average speed (m/s)												
GC-contender	13.30	12.07	13.50	12.45	13.41	12.84	11.09	18.17	12.58	5.40	10.69	12.32
Sprinter	12.94	11.50	13.18	11.95	13.07	12.40	10.37	18.62	12.10	4.48	9.90	11.86
Climber	12.92	11.66	13.13	12.05	13.03	12.45	10.67	17.89	12.18	5.03	10.26	11.93
All-rounder	12.89	11.56	13.11	11.97	13.01	12.39	10.51	18.14	12.11	4.77	10.07	11.87
Time trialist	13.19	11.85	13.41	12.27	13.31	12.69	10.80	18.47	12.41	4.97	10.37	12.16
Mean	13.05	11.73	13.27	12.14	13.17	12.55	10.69	18.26	12.28	4.93	10.26	11.61
95% CI	0.21	0.26	0.20	0.24	0.20	0.22	0.31	0.32	0.23	0.38	0.33	0.26
Estimated finishing time (s)												
GC-contender	401.83	152.06	383.87	436.92	369.70	436.32	127.67	37.20	271.25	286.07	511.96	3414.8
Sprinter	412.87	159.59	393.18	455.35	379.21	451.68	136.63	36.31	282.04	344.54	552.69	3604.1
Climber	413.61	157.36	394.81	451.35	380.38	449.97	132.77	37.78	280.05	307.15	533.58	3538.8
All-rounder	414.57	158.78	395.34	454.43	381.06	452.08	134.79	37.26	281.75	323.82	543.20	3577.0
Time trialist	405.03	154.77	386.37	443.30	372.35	441.32	131.12	36.60	274.92	311.05	527.90	3484.7
Mean	409.58	156.51	390.71	448.27	376.54	446.27	132.60	37.03	278.00	314.53	533.86	3523.9
95% CI	6.40	3.43	5.82	8.80	5.73	7.86	3.83	0.64	5.26	23.97	17.17	84.0
$x_{\mathrm{opt}}$	**0.3818**	**0.4904**	**0.3798**	**0.4449**	**0.3807**	**0.3957**	**0.5920**	**0.3498**	**0.4288**	**0.8781**	**0.6287**	**0.4891**
95% CI	0.0380	0.0198	0.0129	0.0173	0.0130	0.0156	0.0240	0.0145	0.0171	0.0121	0.0268	0.0124

$x_{\mathrm{opt}}$, optimal mass exponent of the power-to-mass ratio.

## 4 Discussion

A method was developed to optimally normalize power output, resulting in a power metric whose magnitude reflects cycling performance. By using this approach, course-specific optimal mass exponents were derived for the power-to-mass ratio, effectively accounting for the variation in estimated time-trial performances among typical professional cyclist profiles across two recent Grand Tour courses. The results further suggest that optimal mass exponents of the power-to-mass ratio are not only course-specific but also vary across individual course sections, depending on the interaction of multiple internal and external factors.

In contrast to many previous studies, which have relied on empirical data from actual performance tests (12, 14, 15, 25), this study estimated course-specific time-trial performances using the power-duration relationship and the power balance equation. This methodological shift offered a key advantage as the investigated performance descriptors, such as velocity and estimated total finishing time, were not affected by differences in pacing strategy between cyclists (26, 27). Moreover, the focus of this study distinguishes it from earlier studies. While classical scaling approaches aim to describe the dependency of a variable on body size (e.g., expressing the dependency of power output on body mass in the form of $P=k\cdot m^{x}$) (28), the method of the current study sought to eliminate the confounding effect of body size on power output to enable fair comparisons across athletes. In doing so, body size independent power metrics optimized for predicting performance over complex time-trial courses were calculated.

Given that an $x_{\mathrm{opt}}$ value closer to one is generally disadvantageous for heavier cyclists, the derived ${\bar{x}}_{\mathrm{opt}}$ values suggest that the stage 21 course of the 2024 Tour de France was less favorable for time trialists compared to lighter GC-contenders or climbers, despite its minimal net elevation change of approximately −3.4 m). This discrepancy can be attributed to the proportion of time spent on uphill vs. downhill sections. If the uphill gradients had been less steep, leading to higher mean section speeds and shorter mean section times, ${\bar{x}}_{\mathrm{opt}}$ would theoretically have been lower. In contrast, the analyzed Giro d’Italia course exhibited a comparatively lower ${\bar{x}}_{\mathrm{opt}}$, suggesting a more favorable scenario for time trialists, despite having significantly more ascent than descent. This interpretation was supported by race outcomes: on stage 7 of the 2024 Giro d’Italia, three time trialists placed in the top 10, with Filippo Ganna (Team Ineos Grenadiers) finishing second. Conversely, on stage 21 of the 2024 Tour de France, the highest-placed time trialist finished 13th. The influence of $m_{\mathrm{equip}}$ was also investigated, showing that a greater proportional contribution of $m_{\mathrm{equip}}$ to $m_{\mathrm{sys}}$ results in lower $x_{\mathrm{opt}}$ values, thus determining the maximum of the $x_{\mathrm{opt}}$ curves. This finding indicates that heavier bicycles pose a relatively greater disadvantage for lighter cyclists than for heavier ones, which supports previous findings on the relative energy cost of uphill cycling (12, 29).

It is important to recognize that actual performance is influenced by the interaction between the course-specific $x_{\mathrm{opt}}$ values and the individual rider’s power profile. Consequently, climbers may occasionally be outperformed on short, steep ascents by all-rounders or even sprinters. Similarly, strong time trialists may serve effectively as domestiques for general classification (GC) contenders on long, moderately steep climbs in stage races. A practical application of the presented method would be to generate power output profiles that are normalized by the course-specific optimal mass exponent, thereby improving the interpretability of power profiles and enabling more accurate rider comparisons tailored to a specific race course or section(s) of the course.

In this study, constant power output time-trial performances were used to determine $x_{\mathrm{opt}}$; however, even in time trials, predicting performance is complex due to the variability in power output distribution along the course (18, 20). Regarding bunch races, the performance predictive ability of the presented method is expected to be lower without additional considerations. Factors such as drafting, energy intake, and in-race dynamics (30–32), all underscore that successful performance in cycling depends not solely on the physiological capacity but also on an optimized pacing strategy, aerodynamic positioning on the bike, nutrition planning, and a good understanding of the race dynamics.

### 4.1 Methodological considerations

By definition, the magnitude of a power metric that accurately reflects performance independently of body mass should remain constant across different body masses at a given speed of travel (see Section 2.5 for details concerning methodology). However, it is crucial to note that the complete mass independence of propulsive power output is technically unattainable due to the inherent non-linearity of the power balance function. As a result, the minimal absolute slope of the regression line is always nonzero. Although this violates the theoretical assumption of perfect mass independence, it does not significantly compromise the method’s accuracy, since the slope of the regression line remains close to zero, resulting in a minimal variation in $P/m^{x_{\mathrm{opt}}}$ (see Figure 3). To quantify this variation, the typical error for consecutive calculations of $P/m^{x_{\mathrm{opt}}}$ was assessed according to the method described by Hopkins (33). Across the constant arbitrary velocities, within the studied body mass range, the typical error was in the order of $10^{-3}$, corresponding to a coefficient of variation of approximately 0.3% for $P/m^{x_{\mathrm{opt}}}$ across all investigated courses. It should also be noted that performance estimation based on power output profiles is inherently limited by fluctuations in athletes’ record power outputs, which can vary both throughout a competitive season and within a single race (5, 6, 34).

### 4.2 Limitations

In this study, we applied a simplified approach for scaling the $C_{d}A$, similar to the work of Sundström et al. (35), for instance. It is important to emphasize, however, that $C_{d}A$ is influenced by several factors, including wind direction, bicycle geometry and configuration, equipment characteristics (e.g., helmet and shoe aerodynamics), and the cyclist’s posture and positioning on the bike. Therefore, for practical implementation of the presented methods and performance metrics, accurate measurements (36, 37), or refined calculations of $C_{d}A$, as well as modeling changes in $C_{d}A$ as a function of wind direction and drafting are necessary [for a detailed explanation see Martin et al. (16) and Blocken et al. (30)]. It is believed that this potential discrepancy between the scaled and actual $C_{d}A$ values was the main reason why the estimated average speeds over the modeled ITTs appeared to be considerably slower than the speed of today’s elite-level cyclists over such efforts. For example, the later winner of the race, Tadej Pogačar (UAE Team Emirates) won the analysed ITT of the 2024 Tour de France (i.e., stage 21) with an average speed of 44.5 km/h, demonstrating an almost 4 km/h positive difference compared to the fastest estimated average speed of 40.7 km/h for this course, which would have resulted in a 25${}^{\mathrm{th}}$ place. Additionally, the normative power profiles used to create the typical cyclist profiles in the current study may no longer reflect the performance capacity of the sport’s top performers, given the rapid advancements in cycling. Since these profiles were derived from averaged historical data, they likely underestimate the capabilities of podium-level athletes.

Another limitation lies in the assumption of a constant power output distribution during the modeled time trials. In this case, power output was not optimally distributed according to terrain profile (18, 20, 27). Instead, a constant mean power output corresponding to the estimated total finishing times was applied across all points of the course for each typical cyclist profile. Additionally, the model did not consider inertia in the direction of travel which may introduce further inaccuracies when analysing courses with numerous turns, or frequent accelerations. As a result of these simplifications, the intermittent expenditure and reconstitution of $W^{\prime }$ were also omitted from the model, likely contributing to the relatively low estimated average speeds across the time-trial courses (38).

Further in-field research is needed to validate whether the magnitude of optimally normalized power output more accurately reflects road cycling performance than previously used metrics (e.g., $W/{\mathrm{kg}}^{0.32}$ and W/kg). Additionally, as the present study focused exclusively on determining ${\bar{x}}_{\mathrm{opt}}$ for male cycling races, it is important to derive and evaluate optimal mass exponents during ITTs for female cyclist profiles to assess potential sex-specific differences.

### 4.3 Practical applications

Despite the various limitations that influenced the results of this study, it remains highly relevant to assess whether the race course profile suits the attributes and abilities of specific cyclists, particularly in the preparation for an upcoming race. Identifying duration-weighted optimal mass exponents of the power-to-mass ratio for complex race courses or key course segments provides valuable insights into the specific demands of a given course in terms of power output relative to body mass. This approach can enhance the interpretation of power output profiles in relation to performance capacity, support the development of targeted training strategies, course profile categorization, and refine cyclist assignment models.

## 5 Conclusions

The present study provides a numerical method for determining the optimal mass exponent of the power-to-mass ratio for optimized cycling performance prediction. The findings demonstrate that optimally normalized power metrics are course-specific. The optimal mass exponent of the power-to-mass ratio increases with steeper inclines but decreases with higher power output, greater equipment mass relative to body mass, and higher headwind velocity relative to the cyclist. These findings underscore the complex interaction between internal and external factors that influence the conversion of power output into cycling speed.

## Data Availability

The original contributions presented in the study are included in the article/Supplementary Material, further inquiries can be directed to the corresponding author/s.

## Appendix

To accurately account for marginal losses of the produced power, Equation 3 can be refined for linear motion and constant velocity v as follows:

(A1) P=(msysg(sin(α)+Crrcos(α))(91+8.7v)10−3+0.5ρ(CdA+Fw)(v+vwindcos(ϕ))2+(91+8.7v)10−3)vEc,

where $F_{w}$ represents the incremental drag area of the spokes, $E_{c}$ is the chain efficiency factor (approximately 0.97) and $(91+8.7v)\cdot 10^{-3}\cdot v$ represents the frictional power loss through the wheel bearings (16).
